# Elephantine Dentals

**Published:** 1879-06

**Authors:** 


### ELEPHANTINE DENTALS.

About six months ago "Conquerer," a large African elephant, one of the herd of elephants belonging to Mr. John

Robinson, of this city, died. The carcass became the property of the Cincinnati Society of Natural History. An opportunity was here afforded for anatomical examination. The dissection was made by Mr. Charles Drury, taxidermist, and a corp of assistants, under the direction of several physicians.

A full report of this autopsy was made by A. G. How, M. D., which was read by him for the Society of Natural History. Of the teeth of the animal, the report says:

"The tusks of the elephant are a distinguished feature of the creature. They spring from the maxillary bones, hence are modifications of incisor teeth. They correspond to the gnawing teeth of the beaver and other rodents. The extinct mammoth had two others which sprang from the lower jaw, and projected forward. The dugong has two in the inferior maxillary which curve downwards, as the tusks of the elephant incline to turn upwards. The tusks of the elephant are largest in the male, and are used as weapons, yet they become useful in breaking down branches, and in uprooting small trees while food is gathered.

The grinders of the elephant are compound teeth, having ridges of dentine on their bruising surfaces. There is but one grinder in each side of either jaw. The tooth springs from the alveolar space in the deep and posterior recesses of the maxillaries, and while growing or developing moves forward, and its thin anterior edge breaks off, so that in the course of several years the entire grinder is lost; but before it is half wasted, another comes along in the rear and occupies the same place and position of the one passing away. In this replacing process, it is not uncommon for an untrue tooth, and part of another to be present in one side of a jaw at the same time. In several respects, the dentition of the elephant is peculiar."

This description is very similar to that given by Owen, except that the latter is rather more explicit; for instance he says: "The crown, of which a great proportion is buried in the socket, and very little more than the grinding surface appears above the gum, is deeply divided into a number of transverse perpendicular plates, consisting each of a body of dentine *coated by a layer of enamel*, and this by the less dense bone-like substance, which fills the interspaces, of the enamel plates, and here more especially merits the name of cement, since it binds together the several divisions of the crowns before they are fully formed and united by the confluence of their bases into a common body of dentine."

These descriptions are similar, except the omission in the former to mention the enamel plates, placed vertically, and abundantly distributed through the tooth.

The description given in this report of the various parts and structures of the elephant, is interesting and instructive.

